# D-dimer assay interference detected by the discrepancy in D-dimer concentrations at different dilutions: a case report

**DOI:** 10.11613/BM.2024.031001

**Published:** 2024-08-05

**Authors:** Marija Milić, Dejana Brkić Barbarić, Iva Lukić, Mirna Kirin, Vikica Buljanović, Vatroslav Šerić

**Affiliations:** 1 Clinical Institute of Laboratory Diagnostics, University Hospital Centre Osijek, Osijek, Croatia; 2 Department of Medicinal Chemistry, Biochemistry and Clinical Chemistry, Faculty of Medicine, University of Osijek, Osijek, Croatia; 3 Clinical Institute of Transfusion Medicine, University Hospital Centre Osijek, Osijek, Croatia; 4 Siemens Healthineers, Siemens Healthcare d.o.o, Zagreb, Croatia; 5 Medical Biochemistry Laboratory, General Hospital Našice, Našice, Croatia

**Keywords:** D-dimer, heterophile antibodies, blood coagulation tests, interference

## Abstract

This case report describes interference from heterophilic antibodies in D-dimer assay. The interference was suspected due to discrepancies between D-dimer concentrations in the original sample and diluted samples, as well as inconsistent clinical findings. The patient’s medical history, laboratory results, and imaging studies were considered in the investigation. Heterophilic antibodies, likely developed during the SARS-CoV-2 infection, were identified as the probable cause of interference. The interference was confirmed through various methods, including dilution studies, blocking heterophilic antibodies, and comparing results with an alternative D-dimer method. This case highlights the importance of recognizing and addressing interference in D-dimer testing, emphasizing the need for collaboration between clinicians and laboratory specialists.

## Graphical abstract

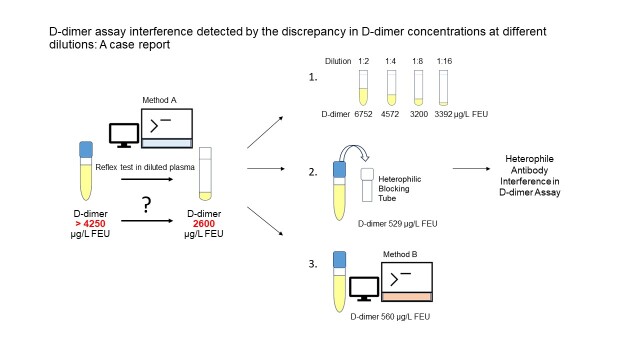

## Highlights

• A case report of interference in D-dimer assay is described

• Heterophile antibodies were identified as the cause of interference

• A practical procedure for detecting interference by heterophilic antibodies is provided

• Collaboration between laboratory specialists and clinicians is essential

## Introduction

D-dimers are the smallest degradation product of fibrin, arising as a result of the activation of the coagulation system and, consequently, the fibrinolytic system (*1*). Elevated concentrations of D-dimers are found in various conditions such as infections, inflammation, pregnancy, trauma, tumors, *etc*. Determining the concentration of D-dimers is important for the diagnosis of deep vein thrombosis, pulmonary embolism, aortic dissection, and disseminated intravascular coagulation (*1*). The methods most used routinely in clinical laboratories are immunoturbidimetric. Since the method for measuring D-dimer concentration is immunochemical, it is susceptible to interferences specific to immunochemical reactions, such as interference by heterophilic antibodies (*2*). These antibodies are directed against animal antibodies and can be found in up to 40% of the population, but they rarely cause problems in immunochemical reactions due to their low affinity and broad reactivity (*3*). However, some heterophilic antibodies can cause interference by binding to antibodies from the reagent, resulting in false positive and rarely false negative results (*3*). Heterophilic antibodies can arise during viral or bacterial infections, after vaccination, in autoimmune and malignant diseases, in contact with animals, animal products, as well as during immunotherapy (*4*). Assay interference with heterophilic antibodies has been well described for hormones, tumor markers, drugs, ferritin, *etc*., while very few cases of interference with D-dimers have been described (*2*, *5*-*12*). It is important to recognize interferences to avoid costly additional tests and unnecessary therapy, prevent delays in diagnosis and treatment, and minimize stress and time loss for the patient.

This is the first documented case of interference in the D-dimers test, detected by the discrepancy in D-dimer concentrations at different dilutions using a reflex test for high D-dimer concentrations. In other published case reports, suspicion of interference arose from clinical doubts about the accuracy of D-dimer results (*7*-*12*).

We consider it important to present this case as it will aid medical biochemists in identifying, recognizing, and confirming interference in D-dimer assays. This information is crucial for proper interpretation and presentation to clinicians.

## Case report

A 65-year-old female patient was hospitalized on April 7, 2022, at the Department of Pulmonology, University Hospital Centre Osijek, 19 days after the onset of the initial symptoms of SARS-CoV-2 infection which was confirmed by a PCR test. The clinical presentation was characterized by a prolonged cough and fatigue. As a comorbidity, she had a known history of arterial hypertension. The patient did not have any previous thromboembolic incidents. At the time she was taking acetylsalicylic acid, trandolapril, and indapamide.

The initially conducted laboratory tests showed mild microcytic anemia with borderline thrombocytosis (hemoglobin 116 g/L, reference interval (RI): 119-157 g/L; platelets 515x10^9^/L, RI: 158-425x10^9^/L), mildly elevated C-reactive protein (CRP) 11.5 mg/L (RI: < 5 mg/L), lactate dehydrogenase (LD) 261 U/L (RI: 130-241 U/L) and creatine kinase (CK) 293 U/L (RI: 17-153 U/L). Fibrinogen was reactively elevated (4.9 g/L, RI: 1.8-3.5 g/L), along with extremely high D-dimer (> 34,536 µg/L FEU, RI: < 500 µg/L FEU) (Innovance D-dimer reagent, BCS XP analyzer, Siemens Healthcare, Marburg, Germany) while prothrombin time (PT) and activated partial thromboplastin time (APTT) were within normal range. Other laboratory tests for kidney and liver function were normal. Given the exceptionally high D-dimer values, a contrast-enhanced chest computed tomography (CT) angiography was performed, ruling out pulmonary thromboembolism. The CT scan described a solid nodular lesion and “ground glass” opacity in the upper lobe of the right lung. Considering the SARS-CoV-2 infection and the elevated D-dimer concentrations, therapeutic doses of low-molecular-weight heparin (LMWH) were initiated. Repeatedly tested D-dimer concentration remained consistently high, and the values are presented in Table 1.

**Table 1:** Time course of D-dimer concentrations during the patient’s follow-up

	**7.4.** **2022.**	**9.4.** **2022.**	**13.4.** **2022.**	**20.4.** **2022.**	**2.5.** **2022.**	**9.5.** **2022.**	**2.6.** **2022.**	**29.6.** **2022.**
D-dimer, µg/L FEU	> 4317	> 4317	> 4317	> 4317	> 4317	> 4250	> 4250	> 4250*
D-dimer (dilution)^†^ µg/L FEU	> 34,536	> 34,536	> 34,536	> 34,536	23,508	12,640	5348	2600*
	**14.7.** **2022.**	**29.9.** **2022.**	**20.12.** **2022.**	**28.12.** **2022.**	**16.2.** **2023.**	**23.2.** **2023.**	**27.4.** **2023.**	**27.6.** **2023.**
D-dimer, µg/L FEU	> 4250*	> 4250*	> 4250*	> 4250*	> 4250*	> 4250*	> 4250*	> 4250*
D-dimer (dilution)^†^ µg/L FEU	3417*	3941*	3797*	3913*	3419*	3077*	3100*	2740*
*Discrepancies of D-dimer in original and diluted samples and were not reported. ^†^D-dimer (dilution) is performed as a reflex test on coagulation analyzer BCS XP using Innovance D-dimer reagent (Siemens Healthineers, Marburg, Germany) when D-dimer concentration is above the measurement range. In this reflex test plasma is diluted 8x with corresponding diluent from the reagent set.								

The patient was discharged in good clinical condition on the eighth day of hospitalization. Over the course of a month, she received therapeutic doses of LMWH with continuous monitoring of her clinical condition (without thromboembolic incidents) and laboratory results under the supervision of transfusion medicine specialist. After a month, D-dimer values showed regression but remained significantly above the reference range (12,640 µg/L FEU). Subsequently, intermediate doses of LMWH were continued for an additional two months.

During this period, the thrombophilia testing was performed, confirming a milder form of hereditary thrombophilia, with heterozygous mutations for plasminogen activator inhibitor-1 (PAI-1) 5G/4G and methylenetetrahydrofolate reductase (MTHFR) C677T along with high activity of coagulation factor VIII (FVIII) 294% (RI: 50-150%). Other tested parameters (antithrombin, protein C, protein S, lupus anticoagulant, immunoglobulins (Ig) G and M cardiolipin antibodies and beta-2 glycoprotein antibodies were within reference interval. For two other genetic markers of thrombophilia (factor V Leiden and factor II G20210A) no mutation was found. Malignant disease was excluded. For the exclusion of significant atherosclerotic and aneurysmal changes in blood vessels, a contrast-enhanced chest CT angiography of the aorta and its branches was performed, showing no abnormalities except for a pericardial effusion with a thickness of 12 mm.

Cardiological assessment, including a 24-hour Holter EKG and echocardiography, revealed minimal pericardial effusion without hemodynamic significance. Due to fatigue symptoms, a thyroid hormone status assessment was conducted, which was within normal limits. Serum protein electrophoresis revealed a monoclonal IgG kappa type, with normal concentrations of IgA, IgM, and IgG, total proteins, and albumin.

A repeated chest CT scan after 3 months showed regression of previously described nodular changes in the upper lobe of the right lung, with chronic “ground glass” changes in the same area. In follow-up laboratory results, approximately 3 months after the start of the treatment, there was continued regression of D-dimer concentrations. However, from June 29, 2022, a discrepancy between the original and diluted samples occurred (Table 1). The dilution protocol performed as a reflex test for high D-dimer values is described in the “Laboratory analyses” section. The concentration of D-dimer has not been reported since then, but only a note that there is a suspicion of interference in the determination of D-dimer. Given the patient’s history and the previous investigations, the application of a reduced prophylactic dose of LMWH was continued.

After the consultation with a specialist in medical biochemistry, due to potential interference in D-dimer determination with rheumatoid factor (RF) and the patient’s rheumatic symptoms (pain in the joints of the hands) RF, antinuclear antibody and anti-cyclic citrullinated peptide antibodies were determined, and they were negative. Patient was examined by a rheumatologist, who concluded that it was degenerative rheumatism without elements of inflammatory rheumatic disease.

A specialist in medical biochemistry conducted additional tests described in the “Laboratory analyses” and “Further investigation” section. Based on the results of these tests, it was concluded that heterophilic antibodies interfere within the D-dimer assay. Considering this information and the fact that previous testing did not reveal the presence of rheumatic (inflammatory), malignant or vascular diseases, thromboprophylaxis with LMWH was discontinued after 14 months. The patient continued therapy with acetylsalicylic acid under continuous monitoring by a transfusion medicine specialist. In the last follow-up on June 27, 2023, the patient was in good clinical condition, with no reported thromboembolic incidents to date. Written informed consent was obtained from the patient for publication of this case report. A publication of this paper was approved by the Ethical Committee of the University Hospital Center Osijek (approval number R1-817/2024).

## Laboratory analyses

The patient’s D-dimer concentration was measured from April 2022 to April 2023 on multiple occasions (Table 1). External quality assessment Croatian Centre for Quality Assessment in Laboratory Medicine (CROQALM) and Randox International Quality Assessment Scheme (RIQAS) was satisfactory during this period. The D-dimer concentration was measured using the Innovance D-dimer reagent on the BCS XP analyzer (Siemens Healthcare, Marburg, Germany). The test was configured to automatically trigger a reflex test in a diluted sample if the initially measured D-dimer concentration exceeded 4100 and up to 4400 µg/L FEU depending on a reagents and calibrators lot. In this reflex test plasma sample is diluted eight times with the corresponding diluent from the reagent set.

In April 2022, the patient’s D-dimer concentration was very high (outside the measurement range > 34,536 µg/L FEU) as a consequence of coronavirus disease 2019 (COVID-19). In May it decreased to 5340 µg/L FEU. However, from June 29, 2022, a discrepancy between the original and diluted samples occurred (Table 1). Since that date, the D-dimer concentration has not been reported but only noted that there is suspicion of interference in D-dimer determination. The specialist in transfusion medicine requested the interpretation of the laboratory findings from the specialist in medical biochemistry because he had to decide on the discontinuation of heparin therapy.

## Further investigation

To clarify and confirm this interference, the concentration of D-dimer was determined at several dilutions, after blocking heterophilic antibodies and with different method. A common strategy to indicate interference is to check for nonlinearity upon dilution (*2*, *4*, *6*). The manufacturer’s diluent and negative plasma with very low D-dimer value were used to prepare the dilutions of the patient’s plasma (Table 2) (*4*, *6*). The lack of linearity was demonstrated: the higher dilution, the lower influence of heterophilic antibodies. However, in the highest dilution this was not the case (Table 2). The cause of this is probably the low concentration range in which the measurement is performed and therefore a larger error is introduced into the calculation. The same pattern is observed for both dilution types (manufacturer’s diluent and negative plasma). Thus, different dilution media did not have a different effect on the binding of heterophilic antibodies. After blocking heterophilic antibodies (Heterophilic Blocking Tube, Scantibodies, Santee, USA) D-dimers were significantly lower, just above the cut-off value (529 µg/L FEU, cut-off 500 µg/L FEU) (Table 3). Additionally, plasma samples obtained on three different dates were sent to another laboratory to determine the D-dimer using a different latex enhanced immunoturbidimetric method and analyzer (reagent D-dimer Beckman Coulter, analyzer DxC 700 AU Beckman Coulter, Brea, USA) (Table 3). In all three plasma samples, the D-dimer determined with the Beckman Coulter reagent were around the cut-off value (500 µg/L FEU) as presented in the Table 3.

**Table 2:** The D-dimer concentrations at different dilutions of patient plasma to check for linearity

**Dilution**	D-dimer (µg/L FEU)*	**D-dimer (µg/L FEU)^†^**
x2	6752	6518
x4	4572	4476
x8	3200	3960
x16	3392	4336
*Dilutions made with manufacturer’s diluent. ^†^Dilutions made with negative plasma *i.e*. plasma with D-dimer < 170 µg/L FEU. All measurements were obtained with the Innovance D-dimer reagent and BCS XP analyzer (Siemens Healthineers, Marburg, Germany).		

**Table 3:** The D-dimer concentrations in plasma samples collected on different dates were determined in the original sample, in dilution, with two different methods and after treatment with the Heterophilic Blocking Tube

		Method A*		**Method B^†^**	
Date	**D-dimer, µg/L FEU**	**D-dimer (diluted)^‡^** **µg/L FEU**	**After HBT treatment** **µg/L FEU**	**D-dimer µg/L FEU**	**After HBT treatment,** **µg/L FEU**
16.2.2023.	> 4250	3419	NP	180	NP
23.2.2023.	> 4250	3077	529	560	620
27.4.2023.	> 4250	3100	NP	430	NP
*Reagent Innovance D-dimer Siemens, analyzer BCS XP (Siemens Healthineers, Marburg, Germany). ^†^Reagent D-dimer Beckman Coulter, analyzer DxC 700 AU (Beckman Coulter, Brea, USA). ^‡^Diluted 8x with Diluent (Siemens Healthineers, Marburg, Germany). HBT - Heterophilic Blocking Tube (Scantibodies, Santee, USA). NP - not performed.					

## What happened? / Solution

Heterophilic antibodies that interfere in immunoassays often develop during viral infections (*3*). Since the patient had COVID-19, heterophilic antibodies likely developed during the COVID-19, causing interference in the D-dimer assay (Innovance D-dimer reagent, BCS XP analyzer, Siemens Healthcare, Marburg, Germany). Interference by heterophilic antibodies in this patient was demonstrated in multiple ways:

In a dilution series, D-dimer concentrations did not match the D-dimer concentration in the original (undiluted) sample (Table 2).

After blocking heterophilic antibodies, the D-dimer concentration decreased approximately sixfold, reaching the cut-off value (Table 3).

D-dimers determined by another method showed significant differences, with the result being at the cut-off value (Table 3).

## Discussion

Interferences in immunochemical methods are unpredictable and diverse. They cannot be completely avoided, regardless of increasingly advanced analytical techniques. Therefore, it is crucial for specialists in medical biochemistry to be well-educated about interferences in immunochemical methods and the ways to detect them. Physicians should also be aware of the limitations of immunochemical methods and consult with biochemists in case of discrepancies between results and clinical findings.

In the previously presented cases of interference, D-dimers were determined using the HemosIL D-dimer HS 500 reagent (Instrumentation Laboratory Company, Bedford, USA), STA-Liatest D-Di Plus (Diagnostica Stago, Asnieres sur Seine, France), and Innovance D-dimer (Siemens Healthcare, Marburg, Germany) (*7*-*12*). In the case presented here, the Innovance D-dimer reagent (Siemens) was used, which, although it contains a heterophile blocking reagent (mice), did not block the antibody present in this patient. However, with the Beckman Coulter D-dimer reagent, D-dimers were around or below the cut-off value (500 µg/L FEU) (Table 3), meaning there was no interference. Although the methods are the same (latex-enhanced immunoturbidimetric assay), the composition of the reagents differs. Antibodies, buffers, heterophile antibody blocking reagents, and other reagent components may vary. One reagent may be susceptible to the action of heterophilic antibodies, while another is not, meaning the blocking of heterophilic antibodies is not effective.

Cevlik *et al.* described a case of interference, unlike all described cases, which was batch specific (*7*). It was observed only in one batch of reagents. Ozbalci *et al.* presented two cases of interference by heterophilic antibodies during COVID-19 and suggested that heterophilic antibodies developed during this viral infection (*8*). In our case as well, the SARS-CoV-2 virus infection preceded the interference, and viral infections are often cited as a cause of developing heterophilic antibodies (*4*).

Zhang *et al.* proposed the use of Fibrin/Fibrinogen Degradation Products (FDP) and the FDP/D-dimer ratio to identify interference (*13*). However, only a small number of laboratories determine FDP along with D-dimers, making this option impractical and unrealistic for routine work.

A limitation of this case report is that not all tests we recommended in the section “What you should/can do in your laboratory to prevent such errors” have been performed. According to Zhang *et al.* FDP/D-dimer ratio should be calculated to confirm interference (*13*). FDP were not measured because this test is not routinely performed in our laboratory nor currently in any laboratory in Croatia.

During routine laboratory work, it is not feasible to reassess every individual D-dimer result, as it would consume a significant amount of time and impose a financial burden on the laboratory. Our case report demonstrates that in some instances, interference can be easily recognized when using method described here, especially when a reflex test with dilution is performed at high concentrations. In such cases, interference can be suspected when there is a discrepancy in concentrations between the original and diluted samples. In addition, the interference in D-dimer determination described here was clarified through good collaboration with a transfusion medicine specialist. Based on this collaboration and evidence of the presence of heterophilic antibodies that interfered with the D-dimer test, it was possible to discontinue LMWH therapy. This spared the patient further unnecessary administration of subcutaneous LMWH, reduced the number of follow-up visits, and ultimately resulted in financial savings.

## What you should/can do in your laboratory to prevent such errors

Interference in the D-dimer assay can arise if the D-dimer concentration of the original sample does not match the dilution, and/or if the D-dimer concentration is inconsistent with the patient’s clinical condition and other findings (radiological and laboratory).

In cases of suspected interference in D-dimer determination, the following steps should be taken:

1. Repeat the analysis to exclude pipetting errors, other technical problems with the analyzer, or human error. Significant variations in repeated measurements (greater than internal control variations) may indicate the presence of heterophilic antibodies.
2. Exclude possible pre-analytical causes of false elevation of D-dimer (hemolysis, clotted sample, lipemia, incorrect patient/sample identification).
3. Repeat the analysis from a new blood sample.
4. Exclude other possible analytical causes of false elevation: elevated RF, monoclonal immunoglobulins, dextrose therapy. It is essential to be familiar with the limitations specified by the manufacturer.
5. After excluding all the mentioned possible causes of elevated D-dimer, determine D-dimers in several dilutions and simultaneously make dilutions of a control sample with D-dimer concentration similar to the tested sample, checking for linearity.
6. Apply the procedure for blocking heterophilic antibodies. Reagents for blocking heterophilic antibodies are available on the market, and every medical-biochemical laboratory should have them.
7. Determine D-dimers by another method. If another method is not available, establish collaboration with other medical-biochemical laboratories using a different method for D-dimer determination. Although very rare, heterophilic antibodies can interfere with another method, but the impact of interference is always different (*3*).
8. Determine FDP in the same plasma sample, if available. Calculate the FDP/D-dimer ratio as it indicates interference according to Zhang *et al.* (*13*).
9. If interference is proven, explain the results to the physician, and do not report the D-dimer result (*4*, *6*).
10. If the routinely used method frequently experiences interference, consider replacing it with a better method, *i.e.*, one in which the manufacturer has taken all available measures to minimize interference.

## Data Availability

All data generated and analyzed in the presented study are included in this published article.
